# Heparin continuous infusion for thromboprophylaxis in pediatric liver transplant recipients

**DOI:** 10.3389/fped.2026.1916517

**Published:** 2026-08-05

**Authors:** Khalid W. Taher, Reema Alhawas, Salma Ansary, Sara Ibrahim, Laian Tarboush, Munirah Alshalawi, Moath Alabdulsalam

**Affiliations:** 1 Pharmaceutical Care Division, King Faisal Specialist Hospital and Research Centre, Riyadh, Saudi Arabia; 2 Pharmacy Practice Department, Alfaisal University, Riyadh, Saudi Arabia; 3 Department of Epidemiology and Biostatistics, College of Public Health and Health Informatics, King Saud bin Abdulaziz University for Health Sciences, Riyadh, Saudi Arabia; 4 Critical Care Medicine Department, King Faisal Specialist Hospital and Research Centre, Riyadh, Saudi Arabia

**Keywords:** heparin continuous infusion, liver transplantation, pediatric, thromboprophylaxis, unfractionated heparin

## Abstract

**Background:**

Vascular thrombosis remains a significant cause of early graft loss and morbidity in pediatric liver transplant (PLT) patients. However, no standardized protocol for thromboprophylaxis exists. This study aimed to evaluate appropriateness of continuous infusion of unfractionated heparin (UFH) prophylaxis in PLT patients.

**Methods:**

This retrospective study was conducted in a pediatric intensive care unit. Patients between the ages of 1 month to 14 years who received prophylactic continuous UFH infusion following PLT were included. The primary outcome was to examine the percentage of patients on intravenous continuous UFH infusion achieving the prophylactic aPTT target levels (40–60 s) in PLT recipients whereas secondary outcomes were time to target, dose titrations, thrombotic events, bleeding complications and safety outcomes. Statistical analyses were performed using Stata software, version 18. For categorical variables, frequencies and percentages were used whereas continuous data was presented as median and interquartile range (IQR).

**Results:**

A total of 47 patients were included, with the majority constituting males (57.4%). Median heparin infusion duration was 55.93 h [38.97–79.97]. All patients achieved prophylactic aPTT target levels, with 89.4% reaching the target within 24 h. Median time to target achievement was 4.68 h [4.00–12.53]. Most patients (63.8%) achieved target prophylactic anticoagulation without dose titration whereas 6.4% required ≥3 titrations. Only 2 patients (4.3%) reported thrombotic complications including one portal vein thrombosis and one hepatic vein thrombosis. Clinically significant bleeding was reported in 7 patients (14.9%), with only one patient requiring surgical re-intervention. Hypotension was observed in 40.4% of patients. Median PICU length of stay was 4 days [3–6].

**Conclusion:**

Postoperative thromboprophylaxis with continuous UFH infusion at an initial dose of 10 units/kg/hr was effective in achieving prophylactic anticoagulation in most PLT recipients. The protocol was associated with low thrombotic events and bleeding complications.

## 1 Introduction

Pediatric liver transplant (PLT) is the definitive treatment for end-stage liver disease (1, 2). In recent years, survival outcomes in PLT have improved significantly due to advancements in surgical procedures and postoperative care, with both 1- and 5-year survival rates remaining above 85% (3, 4). Despite promising outcomes, vascular complications still remain a major concern for early graft loss and morbidity in PLT patients. Children are particularly vulnerable to thrombosis because they have an immature coagulation system (5–7). Bleeding complications due to heparin are reported in approximately 5%–20% of all PLT cases (8). The most frequent complications following PLT are hepatic artery thrombosis (HAT) and portal vein thrombosis (PVT) (9). PVT and HAT incidence vary between 5%–10% and 5%–18%, respectively, with the majority of HAT cases being reported within the first week of transplantation (8, 10, 11). HAT is associated with poor clinical outcomes including graft failure in 60% of cases and mortality rate of 9%–56% (12, 13). Several risk factors for thrombotic complications in PLT have been reported including younger age (<30 months), low body weight (<7 kg), prolonged ischemia time (>450 min), and previous history of abdominal surgeries (14, 15).

Preventing HAT and PVT postoperatively can improve clinical outcomes in PLT patients (16). In clinical practice, a combination of pharmacological and surgical prophylaxis is adopted to reduce thrombosis risk. However, the use of these prophylactic approaches remains controversial (17). Some authors suggest that prophylactic heparin should be given in PLT patients with higher thrombotic risk; however, evidence remains inconsistent (18). Retrospective studies support the use of antiplatelet and anticoagulant prophylaxis in PLT (6). Unfractionated Heparin (UFH) is a fast-acting anticoagulant commonly used to prevent thrombosis in pediatric transplantation (19). It enhances antithrombin III (ATIII) activity and inhibits factor IIA and Xa. Prophylactic use of UFH offers several clinical advantages as it has short half-life and rapid onset and offset of action (20). Colombo et al. reported that UFH significantly reduced venous thrombosis in PLT patients compared to no UFH (2.5% vs. 7.9%, *p* = 0.038) (18).

Despite the effectiveness of UFH for thromboprophylaxis in PLT, no standardized protocol for its use exists. Clinical practice for UFH use in PLT varies considerably between institutions. For instance, there is no consensus regarding initial dosing, dose adjustment, and monitoring approaches (5, 21). To date, there is a paucity of data on optimal starting dose of UFH and titration requirements to achieve and maintain prophylactic aPTT targets. Therefore, this study aimed to evaluate appropriateness of continuous infusion of UFH at starting dose of 10 units/kg/hr for thromboprophylaxis in PLT recipients, and safety profile at a tertiary/quaternary referral center in Saudi Arabia.

## 2 Materials and methods

### 2.1 Study design and population

This retrospective study was conducted in the pediatric intensive care unit (PICU) at a tertiary/quaternary hospital between September 1, 2023, and August 31, 2025. The inclusion criteria were patients between the ages of 1 month to 14 years who underwent PLT and received prophylactic infusion of intravenous UFH during the study period. Patients were excluded if they were either younger than 1 month or older than 14 years. Those who had incomplete medical records related to heparin administration or aPTT monitoring were also ineligible for the study. Patients who received heparin for therapeutic purposes for active thrombosis or had low starting dose (<10 units/kg/hr) were also excluded. Any deaths within 24 h or patients who were withdrawn from care within 24 h were also excluded from analysis.

### 2.2 Anticoagulation protocol and study definitions

UFH was administered as per institutional protocol. Patients were given continuous infusion of intravenous UFH at a starting dose of 10 units/kg/hr. Dose concentration was standardized at 100 units per mL. Anticoagulation effect of UFH was measured using activated partial thromboplastin time (aPTT) every 4 h until target range (40–60 s) was achieved but frequency of measuring aPTT was changed to every 24 h after target aPTT was achieved. Dose adjustments were performed as per predefined aPTT ranges by bedside nurse and treating physician (Table 1). Continuous heparin infusion was defined as postoperative intravenous UFH infusion for at least 24 h initiated within 3 days after PLT. All patients started on heparin infusion were switched to oral aspirin after achieving the target aPTT and tolerated oral intake. The criteria for starting heparin prophylaxis infusion included INR <2 and platelets >50 × 10^9^/L. There were no standard criteria for giving heparin boluses, and it was based on surgeons decisions during the surgery. The prophylactic aPTT target was set at 40–60 s. Clinically significant bleeding was described as any bleeding event during UFH infusion or within 7 days after discontinuation of UFH that resulted in >2 g/dL drop in hemoglobin requiring transfusion, blood product transfusion, or surgical re-intervention. Thrombotic events included documented or suspected PVT, HAT, or DVT during heparin infusion were identified clinically or radiologically within 7 days after UFH infusion. Hyponatremia was defined as serum sodium ≤130 mmol/L. Hypotension was defined as per age-specific systolic blood pressure thresholds including <60 mmHg in neonates, <70 mmHg in infants aged 1–12 months, <70 + (2 × age in years) mmHg in children aged 1–10 years and <90 mmHg in children older than 10 years. Obesity was defined using World Health Organization criterion for children <2 years (22) and the Centers for Disease Control and Prevention calculator for children ≥2 years (23).

**Table 1:** Institution protocol for dose adjustment based on aPTT.

aPTT range (s)	Protocol action
<40	Bolus 50 U/kg, increase rate by 10%
40–60	No change
61–85	Decrease rate by 10%
86–120	Hold 1 h, then restart at 15% lower
>120	Stop infusion and notify physician

### 2.3 Study outcomes

The primary outcome of the study was to examine the percentage (%) of patients on intravenous continuous UFH infusion achieving the prophylactic aPTT target levels (40–60 s) in PLT recipients. Secondary outcomes included time taken to first achieve prophylactic aPTT target levels along with the number and frequency of dose titrations required to maintain the target range. Additional outcomes included PICU length of stay (LOS) and safety of continuous UFH infusion including hypotension, hyponatremia, and hypersensitivity reactions. The occurrence of heparin related thrombotic events and clinically significant bleeding during or shortly after UFH therapy were also assessed. Thrombotic events such as PVT, HAT, or DVT related to subtherapeutic aPTT target levels were evaluated through clinical and radiological documentation while bleeding events were noted based on the need for transfusion, surgical intervention, or discontinuation of heparin as recorded by the treating physicians.

### 2.4 Data collection

Data was collected from Integrated Clinical Information System (ICIS) at our institution. Demographic details of patients and clinical characteristics including primary diagnosis, comorbidities, liver function profile, sodium level, blood pressure, indication for PLT, donor type, graft type, re-transplantation status and previous abdominal surgeries were extracted. Data regarding anticoagulation variables such as UFH initiation time, dose, duration, aPTT values, dose adjustment, and time to aPTT target were also retrieved. Any adverse events if recorded were also noted.

### 2.5 Statistical analysis

Statistical analyses were performed using Stata software, version 18 (StataCorp, College Station, TX, USA). For categorical variables, frequencies and percentages were used whereas continuous data was presented as median and interquartile range (IQR). Normality of data was checked with the Shapiro–Wilk test. PLT patients were divided into 3 age groups for analysis (infant: 1 month—< 12 month, toddler: 12 to <36 months, older child: 36–168 months) were compared using Kruskal–Wallis test for continuous variables and Fisher–Freeman–Halton for categorical variables. Similarly, comparison between patients achieving aPTT target within 24 h and those who achieved after 24 h was done using Mann–Whitney *U*-test for continuous variables and Fisher's exact test for categorical variables. A two-sided *p*-value of <0.05 was set as statistically significant.

### 2.6 Ethics

Ethical approval was obtained from the Institutional Review Board (IRB) prior to commencement of study (IRB# 2251683). The study adhered to the Declaration of Helsinki. IRB waived the need for informed consent due to retrospective study design. All data was anonymized.

## 3 Results

A total of 47 patients were included in the study. Baseline patient characteristics were generally comparable across the age groups. Males represented 57.4% of the overall cohort. Median BMI was 16.05 [14.76–17.76]. Comorbidities were documented in approximately half (57.4%) of the participants. Mechanical ventilation was required more frequently among infants and toddlers compared to older children (overall cohort = 78.7%, *p* = 0.001). Living donor liver transplantation (LDLT) was more common compared to deceased donor liver transplantation (DDLT) (95.7% vs. 4.3%, *p* = 0.715). Re-transplantation was required in only 2 patients. Median baseline INR was 2.40 [1.60–2.80] and 19.1% of patients presented with baseline aPTT values greater than 60 s (Table 2).

**Table 2:** Baseline patient characteristics (*n* = 47).

Variable	Overall (*n* = 47)	Infant (1–<12 months) (*n* = 14)	Toddler (12–<36 months) (*n* = 11)	Older child (36–168 months) (*n* = 22)	*p*-value
Demographics					
Male, *n* (%)	27 (57.4)	12 (85.7)	5 (45.5)	10 (45.5)	0.044
Female, *n* (%)	20 (42.6)	2 (14.3)	6 (54.5)	12 (54.5)	—
BMI, median [IQR]	16.05 [14.76–17.76]	16.03 [14.49–16.71]	16.48 [15.38–18.18]	16.10 [14.49–17.76]	0.820
Obesity, *n* (%)	6 (12.8)	3 (21.4)	0 (0.0)	3 (13.6)	0.385
Comorbidities, *n* (%)	27 (57.4)	9 (64.3)	3 (27.3)	15 (68.2)	0.076
Mechanical ventilation, *n* (%)	37 (78.7)	14 (100.0)	11 (100.0)	12 (54.5)	0.001
Transplant Characteristics					
Donor type—LDLT, *n* (%)	45 (95.7)	14 (100.0)	10 (90.9)	21 (95.5)	0.715
Donor type—DDLT, *n* (%)	2 (4.3)	0 (0.0)	1 (9.1)	1 (4.5)	—
Prior abdominal surgeries, *n* (%)	5 (10.6)	3 (21.4)	0 (0.0)	2 (9.1)	0.236
Baseline Laboratory Values					
INR, median [IQR]	2.40 [1.60–2.80]	2.70 [2.20–2.90]	2.20 [1.60–2.60]	1.95 [1.60–2.70]	0.159
Platelet count, ×10^9^/L, median [IQR]	162 [73–290]	94 [54–255]	236 [114–422]	166 [114–259]	0.080
Hemoglobin, g/L, median [IQR]	95 [89–106]	94.5 [78–103]	92 [90–100]	98 [91–113]	0.216
AST, U/L, median [IQR]	444 [284–678]	415 [293–856]	497 [296–682]	401 [278–576]	0.799
ALT, U/L, median [IQR]	369.4 [245.9–525.8]	353.1 [236.8–669.4]	398.1 [173.8–510.8]	322.3 [266.6–500.3]	0.946
ALP, U/L, median [IQR]	154 [78–213]	121 [66–150]	157 [62–233]	203.5 [146–233]	0.022
Total bilirubin, µmol/L, median [IQR]	53.9 [29.9–101.2]	61.9 [29.9–73.7]	31.9 [11.2–51.4]	57.2 [31.6–126.5]	0.122
Serum creatinine, µmol/L, median [IQR]	17 [14–34]	15 [11–16]	14 [13–21]	34 [23–44]	<0.001
Baseline aPTT, seconds, median [IQR]	47.7 [42.8–54.0]	50.7 [47.6–62.9]	46.7 [43.1–52.9]	45.5 [41.1–49.6]	0.199
Baseline aPTT >60 s, *n* (%)	9 (19.1)	4 (28.6)	1 (9.1)	4 (18.2)	0.508
Baseline serum sodium, mmol/L, median [IQR]	143 [141–146]	147 [145–151]	141 [139–146]	142.5 [141–144]	0.014
MPV, fL, median [IQR] [*n* = 40]	10.6 [9.9–11.7]	10.6 [10.1–11.7] [*n* = 13]	9.8 [9.7–10.8] [*n* = 11]	10.9 [10.6–12.1] [*n* = 16]	0.014

IQR, interquartile range; LDLT, living-donor liver transplantation; DDLT, deceased-donor liver transplantation; MPV, mean platelet volume; aPTT, activated partial thromboplastin time. Continuous variables are presented as median (IQR), and categorical variables as *n* (%). Kruskal–Wallis test was used for continuous variables and Fisher–Freeman–Halton test for categorical variables. Dash (—) indicates no separate statistical comparison performed.

Heparin boluses were given in 46.8% of patients. Median total heparin infusion duration was 55.93 h [38.97–79.97] and did not significantly differ between age groups. Regarding the primary outcome, prophylactic aPTT target levels of 40–60 s were achieved in all patients (100%). Target achievement was defined as the first recorded aPTT value within the prophylactic range of 40–60 s. The majority of patients (89.4%) achieved target aPTT within 24 h of infusion initiation. Patients required a median of 0 [0–1] dose titrations to achieve target prophylactic anticoagulation. Median time to first achievement of target aPTT was 4.68 h [4.00–12.53]. The median heparin dose at the time of target achievement was 10.00 units/kg/hr [10.00–10.90] overall. The majority of patients (63.8%) achieved the prophylactic aPTT target without requiring any dose titration whereas only 6.4% required three or more titrations before reaching the therapeutic goal (Table 3).

**Table 3:** Heparin dosing characteristics and outcomes by age group (*n* = 47).

Variable	Overall (*n* = 47)	Infant (1–<12 months) (*n* = 14)	Toddler (12–<36 months) (*n* = 11)	Older child (36–168 months) (*n* = 22)	*p*-value
Heparin Dosing					
Starting dose, units/kg/hr, median [IQR]	10 [10–10]	10 [10–10]	10 [10–10]	10 [10–10]	0.505
Any heparin bolus given, *n* (%)	22 (46.8)	2 (14.3)	5 (45.5)	15 (68.2)	0.005
Total infusion duration, hours, median [IQR]	55.93 [38.97–79.97]	50.95 [38.98–74.93]	53.00 [31.00–77.97]	57.48 [38.97–86.88]	0.952
Primary Outcome—First Prophylactic aPTT Value (40–60 s)					
aPTT target achieved, *n* (%)	47 (100.0)	14 (100.0)	11 (100.0)	22 (100.0)	—
Target achieved within 24 h, *n* (%)	42 (89.4)	14 (100.0)	10 (90.9)	18 (81.8)	0.181
Target achieved after 24 h, *n* (%)	5 (10.6)	0 (0.0)	1 (9.1)	4 (18.2)	0.181
Secondary Outcomes—Titration Burden and Dose Escalation					
Dose titrations to target, median [IQR]	0 [0–1]	0.5 [0–1]	0 [0–2]	0 [0–0]	0.219
Time to aPTT target achievement, hours, median [IQR]	4.68 [4.00–12.53]	4.38 [4.00–11.50]	6.00 [3.90–17.17]	4.69 [4.00–23.32]	0.737
Dose at target, units/kg/hr, median [IQR]	10 [10–10.9]	10 [10–10.9]	10 [10–10.9]	10 [10–10]	0.425
Target achieved on starting dose (0 titrations), *n* (%)	30 (63.8)	7 (50.0)	6 (54.5)	17 (77.3)	0.203
Target achieved after 1 titration, *n* (%)	9 (19.1)	6 (42.9)	1 (9.1)	2 (9.1)	0.039
Target achieved after 2 titrations, *n* (%)	5 (10.6)	0 (0.0)	2 (18.2)	3 (13.6)	0.291
Target achieved after ≥3 titrations, *n* (%)	3 (6.4)	1 (7.1)	2 (18.2)	0 (0.0)	0.080
Total titrations, all patients, median [IQR]	2 [1–5]	2 [1–5]	3 [1–7]	2 [1–4]	0.431

aPTT, activated partial thromboplastin time. Target achievement was defined as the first single therapeutic aPTT value within 40–60 s. Continuous variables are presented as median (IQR), and categorical variables as *n* (%). Kruskal–Wallis test was used for continuous variables and Fisher–Freeman–Halton test for categorical variables. Dash (—) indicates no formal comparison performed because all patients achieved the target.

Patients who achieved the target after 24 h had comparable total infusion duration compared with those achieving the target earlier (56.47 vs. 54.00 h, *p* = 1.000). Thrombotic events occurred in two patients overall. Bleeding events were reported in 14.9% of patients overall (Table 4). One patient developed PVT and one developed HVT. Hemoglobin drops greater than 2 g/dL requiring transfusion was observed in 10.6% of patients. Blood product transfusion was required in 12.8% of cases, while only one patient required surgical re-intervention for bleeding. Hypotension occurred in 40.4% of patients overall. Hyponatremia occurred in 6.4% of the cohort and was significantly more common among infants (21.4%, *p* = 0.033). No hypersensitivity reactions attributable to heparin were reported. Other adverse events were documented in 12.8% of patients. Median PICU LOS was 4 [3–6] days (Table 5).

**Table 4:** Characteristics and outcomes by timing of aPTT target achievement (*n* = 47 achievers).

Variable	All Achievers (*n* = 47)	Target within 24 h (*n* = 42)	Target after 24 h (*n* = 5)	*p*-value
Patient Characteristics				
Age, months, median [IQR]	24 [10–108]	24 [10–108]	96 [48–132]	0.237
Weight, kg, median [IQR]	10.9 [7.4–25.2]	10.7 [7.4–25.0]	21.0 [14.3–32.5]	0.274
Infant (<12 months), *n* (%)	14 (29.8)	14 (33.3)	0 (0.0)	0.181
Toddler (12–<36 months), *n* (%)	11 (23.4)	10 (23.8)	1 (20.0)	—
Older child (36–168 months), *n* (%)	22 (46.8)	18 (42.9)	4 (80.0)	—
Baseline INR, median [IQR]	2.40 [1.60–2.80]	2.30 [1.60–2.70]	2.90 [1.90–3.50]	0.340
Baseline aPTT, seconds, median [IQR]	47.7 [42.8–54.0]	47.7 [42.9–54.0]	51.5 [39.6–63.9]	0.757
Heparin Characteristics				
Starting dose, units/kg/hr, median [IQR]	10 [10–10]	10 [10–10]	10 [10–10]	0.977
Titrations to target, median [IQR]	0 [0–1]	0 [0–1]	2 [0–2]	0.111
Dose at target, units/kg/hr, median [IQR]	10 [10–10.9]	10 [10–10.9]	10 [8.1–10]	0.177
Total titrations, all patients, median [IQR]	2 [1–5]	3 [1–5]	2 [1–2]	0.735
Total infusion duration, hours, median [IQR]	55.93 [38.97–79.97]	56.47 [38.97–79.97]	54.00 [40.00–57.97]	1.000
Clinical Outcomes				
Thrombotic events, *n* (%)	2 (4.3)	2 (4.8)	0 (0.0)	1.000
Bleeding events, *n* (%)	7 (14.9)	6 (14.3)	1 (20.0)	0.571
Hypotension, *n* (%)	19 (40.4)	18 (42.9)	1 (20.0)	0.635
Hyponatremia, *n* (%)	3 (6.4)	3 (7.1)	0 (0.0)	1.000
PICU length of stay, days, median [IQR]	4 [3–6]	4.5 [3–6]	4 [3–5]	0.473

Target achievement was defined as the first single therapeutic aPTT value within 40–60 s. Continuous variables are presented as median (IQR), and categorical variables as *n* (%). Mann–Whitney *U*-test was used for continuous variables and Fisher's exact test for categorical variables. Dash (—) indicates no separate statistical comparison performed because the *p*-value applies to the overall age-group distribution.

**Table 5:** Safety outcomes and adverse events by age group (*n* = 47).

Variable	Overall (*n* = 47)	Infant (1–<12 months) (*n* = 14)	Toddler (12–<36 months) (*n* = 11)	Older Child (36–168 months) (*n* = 22)	*p*-value
Thrombotic Events					
Any thrombotic event, *n* (%)	2 (4.3)	1 (7.1)	0 (0.0)	1 (4.5)	1.000
Portal vein thrombosis (PVT), confirmed, *n* (%)	1 (2.1)	1 (7.1)	0 (0.0)	0 (0.0)	—
Hepatic vein thrombosis (HVT), confirmed, *n* (%)	1 (2.1)	0 (0.0)	0 (0.0)	1 (4.5)	—
Managed conservatively, *n* (%)	1 (2.1)	1 (7.1)	0 (0.0)	0 (0.0)	—
Requiring surgical re-intervention, *n* (%)	1 (2.1)	0 (0.0)	0 (0.0)	1 (4.5)	—
Bleeding Events					
Any clinically significant bleeding event, *n* (%)	7 (14.9)	2 (14.3)	2 (18.2)	3 (13.6)	1.000
Hb drop >2 g/dL requiring transfusion, *n* (%)	5 (10.6)	2 (14.3)	1 (9.1)	2 (9.1)	—
Blood product transfusion, *n* (%)	6 (12.8)	2 (14.3)	2 (18.2)	2 (9.1)	—
Surgical re-intervention required, *n* (%)	1 (2.1)	0 (0.0)	0 (0.0)	1 (4.5)	—
Multiple criteria met, *n* (%)	2 (4.3)	1 (7.1)	0 (0.0)	1 (4.5)	—
Heparin discontinued due to bleeding, *n* (%)	0 (0.0)	0 (0.0)	0 (0.0)	0 (0.0)	—
Safety Outcomes					
Hypotension, *n* (%)	19 (40.4)	3 (21.4)	7 (63.6)	9 (40.9)	0.106
Lowest SBP during infusion, mmHg, median [IQR]	81 [71–91]	75 [70–78]	71 [68–76]	90 [83–95]	<0.001
Hyponatremia (Na ≤ 130 mmol/L), *n* (%)	3 (6.4)	3 (21.4)	0 (0.0)	0 (0.0)	0.033
Baseline serum sodium, mmol/L, median [IQR]	143 [141–146]	147 [145–151]	141 [139–146]	142.5 [141–144]	0.014
Lowest serum sodium during infusion, mmol/L, median [IQR]	135 [134–137]	134.5 [132–137]	137 [134–138]	135 [134–138]	0.263
Hypersensitivity reaction, *n* (%)	0 (0.0)	0 (0.0)	0 (0.0)	0 (0.0)	—
Other Adverse Events and Clinical Outcomes					
Other adverse events, *n* (%)	6 (12.8)	3 (21.4)	0 (0.0)	3 (13.6)	0.385
PICU length of stay, days, median [IQR]	4 [3–6]	6 [5–11]	4 [3–5]	3 [2–5]	<0.001
Total hospital stay, days, median [IQR]	25 [20–43]	26 [22–61]	22 [16–32]	24.5 [20–35]	0.289

SBP, systolic blood pressure; PVT, portal vein thrombosis; HVT, hepatic vein thrombosis. Hypotension was defined according to age-specific systolic blood pressure thresholds. Hyponatremia was defined as serum sodium ≤130 mmol/L. Continuous variables are presented as median (IQR), and categorical variables as *n* (%). Kruskal–Wallis test was used for continuous variables and Fisher's exact test for categorical variables. Dash (—) indicates no formal statistical comparison performed.

## 4 Discussion

This retrospective study aimed to evaluate appropriateness of continuous intravenous infusion of UFH at a starting dose of 10 units/kg/hr for thromboprophylaxis in PLT patients. The findings show that this protocol is highly feasible for thromboprophylaxis postoperatively as all patients achieved predefined prophylactic aPTT target of 40–60, with the majority reaching this target within 24 h. The median time to first achievement of target aPTT was 4.68 h. However, it should be note that the baseline median aPTT was 47.7 s, which may have contributed to the short time to target attainment. The incidence of thrombotic complications remained low, with only two thrombotic events (4.3%) documented during the study period. Baseline renal profile was within normal limits and liver function tests varied as all patients were candidates for liver transplantation. These lab results did not affect the decision of starting heparin prophylaxis infusion. The only lab criteria influenced the timing of initiating heparin infusion was the baseline INR and platelets count as explained under the protocol of this study. To our knowledge, there are limited published data evaluating standardized continuous UFH thromboprophylaxis in PLT recipients. Thus, our study contributes valuable insight to the existing literature on managing this patient population.

Vascular thrombosis after PLT remains one of the most serious complications associated with graft loss, re-transplantation, and mortality despite major advances in surgical techniques and perioperative management over the last few decades (24, 25). Despite this, optimal thromboprophylaxis strategy in PLT has not yet been clearly established and current international recommendations remain largely based on institutional experience rather than high-quality prospective evidence (18). HAT and PVT remain leading causes of early graft loss. Previous studies reported HAT rates ranging from 5% to 18% and PVT rates between 5% and 10% (7). In the present cohort, overall thrombotic complications occurred in only 4.3% of patients including one confirmed PVT and one HVT. These findings align with previous studies. Colombo et al., in their retrospective study evaluated introduction of 10 units/kg/hr of UFH started 12 h after PLT surgery. Their results showed that venous thrombosis was significantly less frequent in UFH patients compared to those who did not receive UFH (2.5% vs. 7.9%, *p* = 0.038) (18). Similarly, Hardikar et al., in their retrospective study evaluated impact of 10 units/kg/hr of UFH started 24 h postoperatively. Their results also showed that UFH thromboprophylaxis was associated with significantly decreased thrombosis, graft loss, and thrombosis-related mortality (*p* < 0.05 for all) (26).

Some studies have used combination therapy for thromboprophylaxis. For instance, Uchida et al. used intravenous heparin (120 U/kg/day) during the first postoperative week followed by oral dipyridamole (4 mg/kg/day). They reported HAT in 6.7% (27). McLin et al. propose that fresh frozen plasma should be used in combination with heparin postoperatively to maintain adequate antithrombin III (ATIII) which ensures heparin efficacy (28). Lemoine et al. found that a risk-adjusted post-LT anticoagulation protocol resulted in an incidence of HAT and PVT of 5.5% each, with few bleeding complications (29). Similarly, Borst et al. reported that prophylactic UFH was not associated with an increased risk of bleeding (6).

In the present study, all patients achieved target anticoagulation with 89.4% achieving the prophylactic target within 24 h of infusion initiation. However, only 10.6% required more than 24 h to reach the target range. The median time to anticoagulation target was 4.68 h and more than half of patients (63.8%) required no dose titrations. These findings suggest some variability of heparin responsiveness in PLT recipients. Several physiological mechanisms may explain this variability. Children undergoing PLT possess a unique rebalanced hemostatic system characterized by simultaneous reductions in procoagulant and anticoagulant factors (30). Werner et al. demonstrated that despite prolonged conventional coagulation assays such as INR and aPTT, children after PLT may remain relatively hypercoagulable because recovery of prothrombotic pathways exceeds recovery of natural anticoagulants (8). Even after transplantation, this prothrombotic tendency persists. For instance, van Dievoet et al. found that even three months after transplantation, coagulation profiles had not fully normalized as most patients continued to show resistance to thrombomodulin despite normalized liver function (31). Such data imply that PLT patients are intrinsically prone to vascular thrombosis.

In the present study, baseline aPTT values exceeded 60 s in almost one-fifth of the cohort before UFH initiation. Despite this apparent laboratory coagulopathy, thrombotic events still occurred. This observation further supports the modern concept that conventional coagulation tests inadequately predict thrombosis risk after PLT. Borst et al. reported that routine laboratory assays poorly predicted bleeding and thrombotic complications in PLT recipients (6). Consequently, reliance solely on INR or aPTT to estimate coagulation balance may be misleading in PLT patients. This study also found that infants and toddlers had numerically shorter time to target anticoagulation compared with older children, although the difference was not statistically significant. This may be explained by the fact that the coagulation profile of the older children is closer to that of adults than it is to that of infants and toddlers. Therefore, this finding should be interpreted cautiously. It may still reflect known developmental differences in coagulation physiology, antithrombin activity, and heparin pharmacokinetics in infants and toddlers (32) but a larger study would be needed to confirm whether age-related effects are seen in UFH response. Previous studies however have shown that low postoperative antithrombin activity is associated with increased vascular thrombosis after PLT (33).

The safety profile observed in the current cohort was generally acceptable. Clinically significant bleeding occurred in 14.9% of patients while only one patient required surgical reintervention. Although bleeding remains a major concern during postoperative anticoagulation, these rates compare favorably with previously reported PLT studies. Borst et al. reported major bleeding complications in approximately 20.7% of pediatric transplant procedures (6). Similarly, published literature has documented bleeding rates ranging between 5% and 20% among PLT recipients receiving anticoagulation (7). Hyponatremia was minimal and occurred only in 6.4% of the cohort. It was not clinically significant as none of the patients required more than administering sodium supplements.

This study provides valuable real-world data on UFH thromboprophylaxis in PLT recipients. Implications of current findings may help inform clinical practice guidelines. Despite this, there are several limitations of the present study that should be considered prior to interpreting these findings. The small sample size limits statistical power of analysis to identify subgroup differences. The definition of target anticoagulation reflects early response which pertains to the first aPTT value within range and not sustained control over time. Another limitation of this study is the retrospective study design and single-center data. These findings may introduce bias due to reliance on electronic medical records and limit generalizability of findings to other institutions. The study also did not have a control group to compare outcomes. Additionally, data on concomitant medications such as antithrombotic or prothrombotic agents were not collected. This limitation may have influenced some of the laboratory values reported and should be accounted for in future studies.

## 5 Conclusion

The findings of the present study showed that prophylactic intravenous infusion of UFH is feasible for achieving target anticoagulation levels in PLT patients. All patients achieved aPTT target, with the majority achieving this target within 24 h. Thrombotic complications and adverse events were uncommon in present study. Larger multicenter studies are further needed to establish evidence-based thromboprophylaxis guidelines.

## Data Availability

The raw data supporting the conclusions of this article will be made available by the authors, without undue reservation.
